# Artificial intelligence-based diagnostic model for schizophrenia in individuals living with HIV

**DOI:** 10.3389/fpsyt.2026.1709861

**Published:** 2026-03-09

**Authors:** Junzhi Chen, Tongping Ren, Jianjian Li, Meiling Li, Xiongjun Li, Chunyang Hu, Zhongliang Jiang, Xiaolin He, Youwang Lu

**Affiliations:** 1 College of Nursing, Dali University, Dali, Yunnan, China; 2 School of Public Health, Kunming Medical University, Kunming, Yunnan, China; 3 Yunnan Provincial Hospital of Infectious Disease/Yunnan AIDS Care Center, Kunming, Yunnan, China; 4 School of Public Health, Dali University, Dali, Yunnan, China

**Keywords:** diagnostic model, hematological parameters, HIV/AIDS, machine learning, schizophrenia

## Abstract

**Background:**

Schizophrenia is one of the most prevalent severe mental disorders among people living with HIV (PLWH). Delayed diagnosis and misdiagnosis contribute to poor prognosis and substantial economic burden in this population. However, there are currently no validated diagnostic models available for schizophrenia in PLWH.

**Methods:**

PLWH attending annual follow-ups at Yunnan Provincial Hospital of Infectious Diseases/Yunnan AIDS Care Center were enrolled. Hematological parameters were compared between PLWH with schizophrenia (HIV-Scz) and those without (HIV-non-Scz) and diagnostic models were constructed using six machine learning algorithms. Model performance was evaluated comprehensively using area under the curve (AUC), accuracy, F1 score, recall, precision, and decision curve analysis. SHapley Additive exPlanations (SHAP) were applied to determine the relative importance of each feature.

**Results:**

A total of 186 participants were included in this study, including 62 with clinically confirmed schizophrenia who were receiving antipsychotic treatment at the time of blood sampling. Compared with the HIV-non-Scz group, the HIV-Scz group exhibited significant differences across multiple hematological parameters. Six machine learning models constructed using 28 routine blood parameters demonstrated diagnostic capability, among which the Lasso regression model achieved the best overall performance, with mean AUC (0.966 ± 0.016), F1-score (0.839 ± 0.067), and accuracy (0.897 ± 0.037), together with favorable precision (0.867 ± 0.061) and recall (0.821 ± 0.111). Decision curve analysis indicated that this model provided a higher net benefit within clinically relevant threshold probability ranges. Furthermore, SHAP analysis identified PDW, MPV and MCV as the most influential features contributing to model predictions.

**Conclusion:**

Routine hematological parameters may serve as potential diagnostic biomarkers for schizophrenia in PLWH, although medication-related effects in treated patients cannot be excluded.

## 1 Introduction

Schizophrenia is a complex mental disorder with profound and long-term impacts on morbidity, mortality, and quality of life (1, 2). Globally, people living with HIV (PLWH) are at substantially higher risk of developing severe psychiatric disorders, including schizophrenia (3–5). Studies in Western populations have reported that PLWH are several times more likely to receive a schizophrenia diagnosis than HIV-negative individuals (6–9). The mortality rate of schizophrenia among PLWH is up to 25.8% (10), and the situation in China is also concerning. Although epidemiological data remain limited, emerging evidence indicates that the prevalence of psychiatric comorbidities among Chinese PLWH is increasing, and schizophrenia constitutes one of the most common severe mental disorders in this population (11–13).

Clinical outcomes among individuals with schizophrenia are often adversely affected by delayed diagnosis and misdiagnosis. Currently, diagnosis relies heavily on clinical assessment based on standardized criteria systems like DSM-5 or ICD-10 (14, 15). However, schizophrenia exhibits considerable heterogeneity in clinical presentation and disease trajectory (16). Furthermore, symptom overlap with other psychiatric disorders frequently leads to diagnostic errors (17). Additionally, schizophrenia-related symptoms in people with HIV may overlap with HIV-associated neurocognitive disorders or drug-related psychiatric symptoms. This diagnostic ambiguity complicates differential diagnosis and increases the risk of missed or delayed diagnosis.

Given these challenges, there is an urgent need for objective and reliable diagnostic tools to improve early detection and intervention. In this context, increasing attention has been directed toward potential blood-based biomarkers that reflect disease pathophysiology. Complete blood count (CBC) is a simple and routinely performed laboratory test widely used to assess the composition and concentration of blood cell components, and it can provide indirect information on conditions such as infection, anemia, dehydration, and malnutrition (18). Studies have demonstrated that blood-based inflammatory biomarkers can serve as low-cost indicators for mental health screening in people living with HIV (19). In recent years, machine learning (ML) and artificial intelligence (AI) approaches have demonstrated substantial promise in medical diagnostics by integrating complex clinical, laboratory, and demographic data to identify latent patterns that are difficult to detect using conventional assessment methods (20, 21). Machine learning algorithms have been applied in various forms to the diagnostic research of schizophrenia and related psychiatric disorders. For example, Ogur et al. developed diagnostic models for schizophrenia based on peripheral blood biomarkers, reporting areas under the receiver operating characteristic curve (AUC) of 0.96 and 0.95 for the XGBoost and random forest models, respectively, indicating favorable diagnostic performance (22). Zhu X et al. analyzed peripheral blood gene expression datasets from patients with schizophrenia and employed machine learning approaches, including random forest, least absolute shrinkage and selection operator (Lasso) regression, and support vector machine–recursive feature elimination (SVM-RFE), to identify immune-related hub genes associated with schizophrenia (23). Kozyrev et al. constructed schizophrenia diagnostic models based on peripheral inflammatory biomarkers using logistic regression, deep neural networks, decision trees, support vector machines, and k-nearest neighbor algorithms (24). Zhu L et al. examined messenger RNA (mRNA) expression levels in peripheral blood and applied multiple machine learning algorithms—including artificial neural networks, XGBoost, support vector machines (SVM), decision trees, and random forest—to discriminate patients with schizophrenia from healthy controls (25). Wagh VV et al. developed SVM and prediction analysis for microarrays (PAM) models using differentially expressed genes as features to improve the diagnostic accuracy of psychiatric disorders (26). Collectively, these predictive models may assist clinicians in making more timely and accurate diagnostic decisions and have the potential to improve clinical outcomes in high-risk populations (27). Notably, to date, no studies have applied machine learning or AI models specifically to predict schizophrenia among people living with HIV. Therefore, in this study, we aimed to develop and evaluate AI-driven diagnostic models for schizophrenia among PLWH by leveraging routine hematological and clinical parameters. We further investigated the interpretability of the models to identify key features contributing to schizophrenia risk, with the ultimate goal of providing a clinically applicable and objective diagnostic tool for this vulnerable population.

## 2 Materials and methods

### 2.1 Data sources

This study enrolled 62 PLWH with schizophrenia (HIV-Scz group) and 124 PLWH without schizophrenia (HIV-non-Scz group) who visited the Yunnan Provincial Hospital of Infectious Disease between January 1, 2018, and December 31, 2024. Schizophrenia was diagnosed according to the ICD-10 criteria (F20-F20.9). The identifying information (names, phone numbers, and home addresses, etc.) has been anonymized to ensure patient confidentiality. This study has been approved by the ethics committee of Yunnan Provincial Hospital of Infectious Disease (No. 201943).

Patients were enrolled in this study according to the inclusion and exclusion criteria. Inclusion criteria: (1) age ≥ 18 years; (2) confirmed diagnosis of HIV/AIDS; (3) availability of complete hematological data from the first blood test during hospitalization; and (4) confirmed diagnosis of schizophrenia for participants in the HIV-Scz group. Exclusion criteria: (1) comorbid severe psychiatric disorders (e.g., bipolar disorder or major depressive disorder); (2) presence of serious organic brain diseases or acute somatic infections; (3) concurrent opportunistic infections or co-infection with hepatitis B virus or hepatitis C virus; and (4) missing essential clinical data.

### 2.2 Data collection

In this study, patients’ clinical data, including demographic characteristics, medical history, and laboratory test results, were retrieved from electronic medical records. Hematological parameters were subsequently used as candidate predictors for schizophrenia among individuals living with HIV. Hematological parameters were obtained from CBC analyses performed using the XN-1000 hematology analyzer (Sysmex, Kobe, Japan), including platelet count (PLT), plateletcrit (PCT), white blood cell count (WBC), monocyte count (MON#), monocyte percentage (MON%), lymphocyte count (LYM#), lymphocyte percentage (LYM%), neutrophil count (NEU#), neutrophil percentage (NEU%), mean corpuscular hemoglobin concentration (MCHC), platelet distribution width (PDW), hemoglobin concentration (HGB), red blood cell distribution width-coefficient of variation (RDW-CV), mean platelet volume (MPV), basophil count (BAS#), basophil percentage (BAS%), eosinophil count (EOS#), eosinophil percentage (EOS%), mean corpuscular volume (MCV), mean corpuscular hemoglobin (MCH), hematocrit (HCT), red blood cell count (RBC), and red blood cell distribution width-standard deviation (RDW-SD).

Additionally, inflammatory indicators, including neutrophil-to-lymphocyte ratio (NLR), monocyte-to-lymphocyte ratio (MLR), platelet-to-lymphocyte ratio (PLR), systemic immune-inflammation index (SII), systemic inflammation response index (SIRI) were obtained within six hours of sample collection. The calculation formulas for the indices are as follows:

SII = (Neutrophil count × Platelet count)/Lymphocyte count;
SIRI = (Neutrophil count × Monocyte count)/Lymphocyte count;
PNI = 10 × Albumin (g/dL) + 0.005 × Lymphocyte Count (/mm³).

### 2.3 Development of the diagnostic models

Model development and performance evaluation were conducted using five-fold cross-validation on the entire dataset. All 28 blood parameters were initially included. In each iteration, one fold was held out as an independent test set, while the remaining four folds were used as the training set. Within the training set, an inner cross-validation procedure was performed exclusively for feature selection and hyperparameter tuning. Model performance was assessed based on the average cross-validation results. Six machine learning algorithms were employed to construct schizophrenia diagnostic models: logistic regression, Lasso regression, SVM, random forest, extreme gradient boosting (XGBoost), and gradient boosting machine (GBM). These algorithms have been widely applied in medical diagnosis and disease risk prediction studies and are capable of providing robust predictive performance across different types of clinical data.

Feature selection plays a critical role in model development by removing redundant variables and reducing noise, thereby improving model robustness and interpretability and enabling results that better reflect real-world conditions. In this study, model-specific feature selection strategies were applied for different machine learning algorithms.

For Lasso and logistic regression models, the optimal regularization parameter (λ) was determined using 5-fold cross-validation, and features with non-zero coefficients at the value of λ that minimized the cross-validated error (λ_min) were retained for subsequent analyses. Feature selection for the SVM model was performed using recursive feature elimination (RFE) implemented in the caret package (version 7.0-1). For the random forest classifier and GBM model, feature selection was conducted using the Boruta algorithm (version 8.0.0), a wrapper-based method that identifies relevant variables by comparing their importance with randomized shadow features.

For XGBoost, feature importance was quantified using the Gain metric, which reflects the contribution of each feature to the reduction of the loss function during tree splitting. Features with Gain values greater than the mean Gain across all variables were retained as informative features for subsequent model training. All feature selection procedures were performed exclusively on the training dataset to avoid information leakage.

To reduce the risk of overfitting and improve model robustness, five-fold cross-validation was applied to all models within the entire dataset. Specifically, the entire dataset was randomly divided into five subsets. In each iteration, four subsets were used for model training, and the remaining subset was used for validation. This process was repeated five times. Feature combinations and hyperparameters were optimized based on comprehensive performance metrics during the cross-validation phase. The optimal model was then retrained.

Predictive performance was optimized through a tuning process, with the parameter settings and tuning strategies for each model as follows:

Lasso regression employed L1 regularization, with the regularization parameter λ optimized via five-fold cross-validation. The search range for λ was set from 10^−4^ to 10¹, with 100 logarithmically spaced candidate values. The λ value minimizing the cross-validation error was selected as the optimal parameter.

Logistic regression was constructed using default parameters without additional hyperparameter tuning.

SVM used a linear kernel, with the penalty parameter C optimized via grid search combined with five-fold cross-validation. Candidate C values ranged from 2^−^² to 2^6^ (0.25 to 64) with logarithmic increments (base 2), testing a total of nine values to balance model complexity and classification tolerance.

Random forest optimized the key parameter mtry (the number of features randomly selected at each node split) via grid search and five-fold cross-validation. The search range was set from 1 to 19 (step size = 1), corresponding to the total number of features. The value minimizing out-of-bag error or cross-validation error was selected, balancing the predictive power of individual trees with overall model generalization.

XGBoost tuned core hyperparameters using grid search with five-fold cross-validation. The parameter space was defined as follows: number of iterations (nrounds) = 50 and 100; maximum tree depth (max_depth) = 2–3; learning rate (eta) = 0.05, 0.1, 0.3; column sampling ratio (colsample_bytree) = 0.8; row sampling ratio (subsample) = 0.8; minimum child weight (min_child_weight) = 1. The optimal parameter combination was determined based on minimizing cross-validation error.

GBM also applied grid search with five-fold cross-validation for parameter optimization. For tree structure parameters, the number of boosting iterations (n.trees) was set to 100, 200, and 500, the maximum tree depth (interaction.depth) to 1, 3, and 5, and the minimum number of observations in terminal nodes (n.minobsinnode) was set to 10 and 20. The learning rate (shrinkage) was tuned between 0.01 and 0.1 to control the contribution of each tree. The optimal configuration was determined according to the principle of maximizing cross-validated AUC (or minimizing cross-validation error).

### 2.4 Evaluation of the models

The performance of the models was quantitatively evaluated using AUC, accuracy, precision, recall, and F1 score. Decision curve analysis (DCA) was performed to assess the clinical application value of each model. Furthermore, Shapley Additive Explanations (SHAP) analysis was employed to interpret the contribution of individual variables to the predictive model.

### 2.5 Statistical analysis

Continuous variables were presented as the mean ± standard deviation (mean ± SD) for normally distributed data or as the median with interquartile range [M (IQR)] for non-normally distributed data. Categorical variables were summarized as frequencies and percentages (%). Comparisons between two groups were performed using the *t*-test for normally distributed continuous variables, the Wilcoxon rank-sum test for non-normally distributed continuous variables, and the chi-square (χ²) test for categorical variables. *P*-values were adjusted using the Bonferroni correction method, and a two-sided P value < 0.05 was considered statistically significant.

All statistical analyses were conducted using R software (version 4.1.2), and the entire model development workflow was implemented within the caret framework. Model-specific feature selection procedures were implemented as described in Section 2.3 and were not uniformly applied across all algorithms. First, multicore parallel computing was enabled using the doParallel package (version 1.0.17) to improve computational efficiency. Next, hyperparameter tuning and model construction were carried out under the caret framework using 5-fold cross-validation for logistic regression, SVM, Lasso regression based on glmnet (version 4.1-8), random forest based on randomForest (version 4.7-1), XGBoost based on xgboost (version 1.6.0.1), and GBM based on gbm (version 2.2.3). Finally, model discriminative performance was evaluated using the pROC package (version 1.18.5), and SHAP analysis was performed using the shapviz package (version 0.9.7) to enhance model interpretability.

## 3 Results

### 3.1 Demographic, clinical, and laboratory characteristics

**Figure 1:**
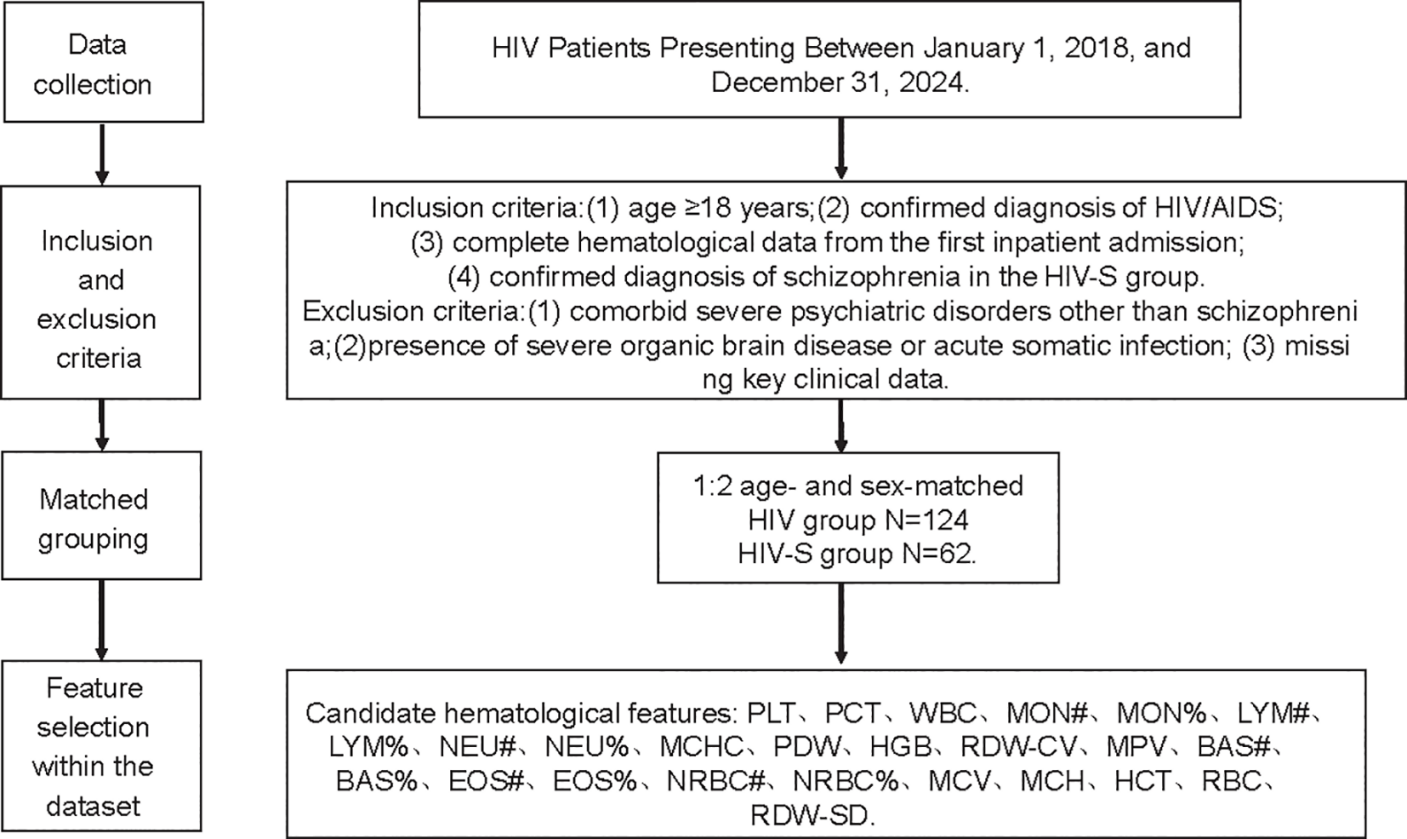
Flowchart of study design and data collection.

As shown in **Figure 1**, the patients were enrolled according to strict inclusion and exclusion criteria. Ultimately, a total of 186 individuals were included in this study, comprising 62 PLWH with schizophrenia (HIV-Scz group) and 124 PLWH without schizophrenia (HIV-non-Scz group). **Table 1** summarizes the baseline demographic and hematological characteristics of the HIV-infected patients with schizophrenia (HIV-Scz) and those without schizophrenia (HIV-non-Scz). No significant differences were observed between the two groups in terms of age or sex distribution (all *P* > 0.05), indicating good baseline comparability. Compared with the HIV-non-Scz group, the HIV-Scz group exhibited significantly lower erythrocyte-related parameters, including mean corpuscular volume (MCV), hemoglobin (HGB), hematocrit (HCT), mean corpuscular hemoglobin (MCH), and mean corpuscular hemoglobin concentration (MCHC) (all *P* < 0.001). In addition, platelet distribution width (PDW) was significantly reduced in the HIV-Scz group (*P* < 0.001). Regarding immune cell–related indices, the HIV-Scz group showed significantly higher eosinophil percentage (EOS%) and monocyte percentage (MON%), whereas the absolute lymphocyte count (LYM#) was significantly lower (all *P* < 0.05). No significant differences were observed between the two groups in platelet count, plateletcrit, or composite inflammatory indices, including the neutrophil-to-lymphocyte ratio (NLR), platelet-to-lymphocyte ratio (PLR), systemic immune-inflammation index (SII), and systemic inflammation response index (SIRI).

**Table 1:** Demographic, clinical, and laboratory characteristics of the participants.

Parameter	HIV-non-Scz (n=124)	HIV-Scz (n=62)	*P*-value	*P*-adjusted
male	78.00	39.00	1.00	1.00
age	43.50 [39.00, 50.00]	43.50 [39.00, 50.00]	1.00	1.00
LYM%	35.28 ± 9.49	32.63 ± 12.98	0.12	1.00
MPV	9.71 ± 1.64)	10.31 ± 1.09	0.01	0.54
NEU%	54.89 ± 9.60)	55.31 ± 13.40	0.81	1.00
RBC	4.27 ± 0.70)	4.19 ± 0.86	0.52	1.00
PLT	216.00 [161.25, 254.50]	205.00 [154.50, 240.00]	0.20	1.00
MCHC	346.65 [340.72, 353.60]	338.00 [328.25, 346.00]	<0.001	<0.001
PDW	16.65 [14.38, 17.40]	11.10 [10.10, 12.73]	<0.001	<0.001
NEU#	3.01 [2.43, 3.70]	2.74 [1.89, 3.58]	0.07	1.00
LYM#	1.90 [1.52, 2.38]	1.54 [1.25, 1.87]	<0.001	0.02
MCH	35.50 [33.10, 40.95]	32.05 [30.05, 34.82]	<0.001	<0.001
MCV	102.40 [96.47, 115.95]	95.75 [90.78, 103.40]	<0.001	<0.001
RDW-CV	13.00 [13.00, 14.00]	13.00 [13.00, 14.00]	0.21	1.00
HGB	153.00 [143.75, 165.00]	134.00 [120.50, 149.00]	<0.001	<0.001
WBC	5.60 [4.80, 6.54]	4.90 [4.00, 6.43]	0.02	1.00
EOS#	0.08 [0.05, 0.12]	0.11 [0.06, 0.17]	0.01	0.33
MON#	0.40 [0.34, 0.50]	0.41 [0.33, 0.56]	0.35	1.00
BAS%	0.46 [0.30, 0.69]	0.30 [0.20, 0.50]	0.02	1.00
EOS%	1.40 [0.80, 2.30]	2.30 [1.30, 3.40]	<0.001	0.01
HCT	43.90 [41.48, 47.80]	40.20 [35.80, 44.00]	<0.001	<0.001
BAS#	0.02 [0.01, 0.03]	0.02 [0.01, 0.02]	0.29	1.00
PCT	0.20 [0.16, 0.23]	0.20 [0.16, 0.24]	0.98	1.00
MON%	7.30 [6.18, 8.40]	8.40 [7.03, 9.88]	<0.001	0.01
RDW-SD	49.00 [46.00, 54.00]	48.00 [44.00, 52.75]	0.07	1.00
NLR	0.08 [0.06, 0.12]	0.09 [0.05, 0.16]	0.93	1.00
MLR	0.01 [0.01, 0.02]	0.01 [0.01, 0.02]	0.07	1.00
PLR	6.15 [4.57, 7.61]	6.25 [4.26, 8.68]	0.55	1.00
SII	326.87 [224.96, 447.85]	318.80 [215.16, 534.71]	0.63	1.00
SIRI	0.62 [0.44, 0.92]	0.79 [0.34, 1.33]	0.19	1.00

HIV, human immunodeficiency virus; LYM%, lymphocyte percentage; MPV, mean platelet volume; NEU%, neutrophil percentage; RBC, red blood cell count; PLT, platelet count; MCHC, mean corpuscular hemoglobin concentration; PDW, platelet distribution width; NEU#, absolute neutrophil count; LYM#, absolute lymphocyte count; MCH, mean corpuscular hemoglobin; MCV, mean corpuscular volume; RDW-CV, red cell distribution width–coefficient of variation; HGB, hemoglobin; WBC, white blood cell count; EOS#, absolute eosinophil count; MON#, absolute monocyte count; BAS%, basophil percentage; EOS%, eosinophil percentage; HCT, hematocrit; BAS#, absolute basophil count; PCT, plateletcrit; MON%, monocyte percentage; RDW-SD, red cell distribution width–standard deviation; NLR, neutrophil-to-lymphocyte ratio; MLR, monocyte-to-lymphocyte ratio; PLR, platelet-to-lymphocyte ratio; SII, systemic immune-inflammation index; SIRI, systemic inflammation response index; SD, standard deviation; IQR, interquartile range; p adjusted, adjusted p-value using Bonferroni correction.

### 3.2 Development and evaluation of artificial intelligence-driven diagnostic models

**Figure 2:**
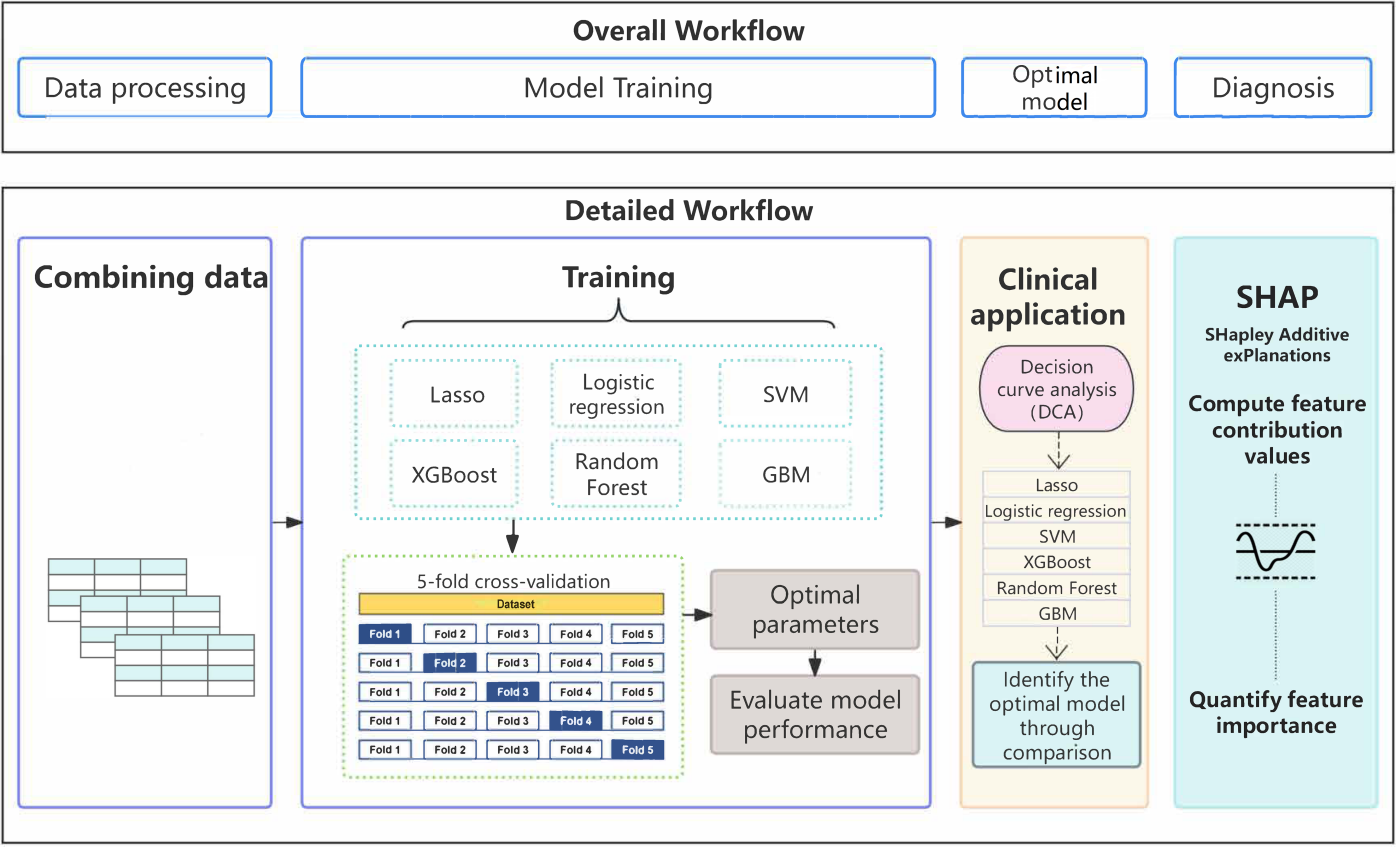
Flowchart of diagnostic model development and validation.

**Figure 3:**
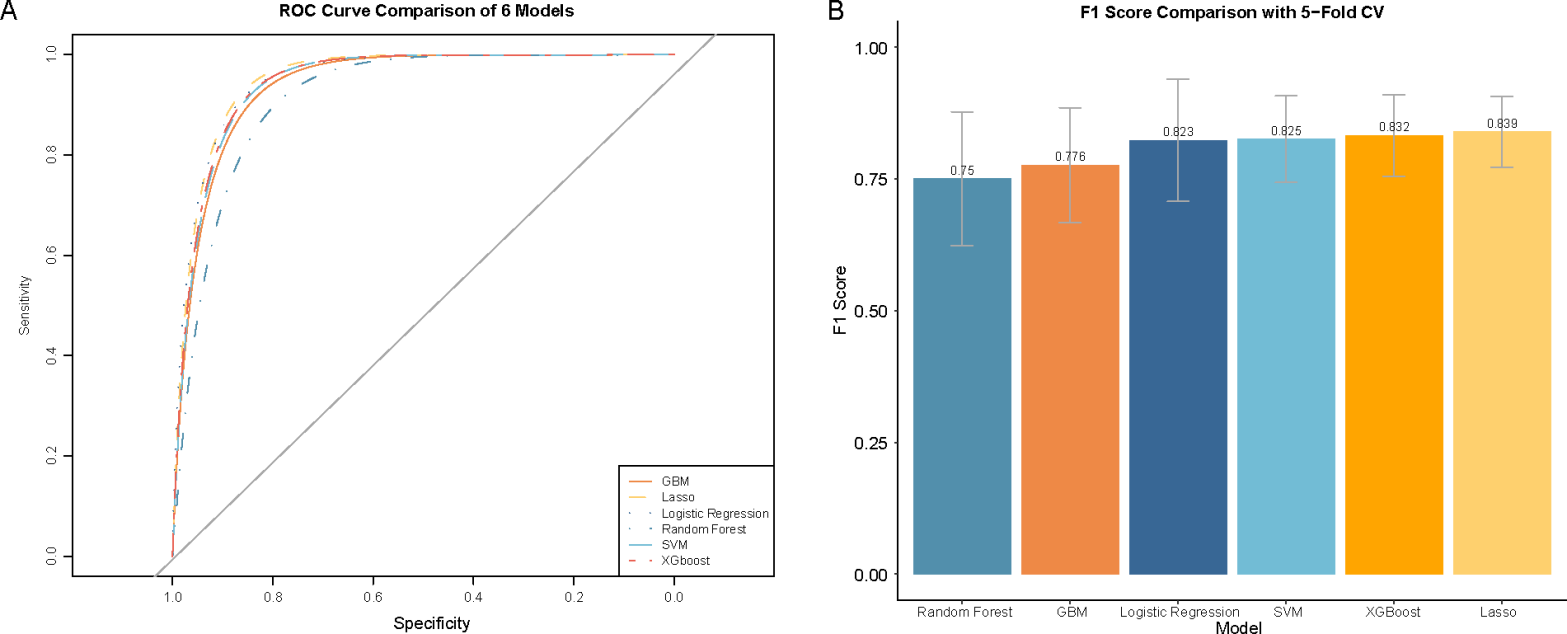
Comparison of diagnostic performance of six ML models for schizophrenia in PLWH. **(A)** Receiver operating characteristic (ROC) curves of different ML models. **(B)** F1 scores of different ML models. Abbreviation: ROC, receiver operating characteristic.

Twenty-eight hematological parameters were incorporated into six machine learning algorithms to develop diagnostic models (**Figure 2**). All six models demonstrated good discriminatory ability based on five-fold cross-validation (**Figure 3**). Among them, the Lasso model achieved the best overall performance, with the highest mean AUC (0.966 ± 0.016), F1-score (0.839 ± 0.067), and accuracy (0.897 ± 0.037), together with favorable precision (0.867 ± 0.061) and recall (0.821 ± 0.111). The SVM, logistic regression, and XGBoost models also demonstrated strong discriminative ability, with AUCs ranging from 0.944 to 0.954 and balanced performance across multiple metrics. In contrast, the random forest and GBM models showed relatively lower overall performance, particularly in recall and F1-score, indicating comparatively weaker classification capability. Model performance was evaluated by calculating the mean values across the five folds, which were used to assess the diagnostic efficacy of each model. The corresponding standard deviations were also reported to reflect model stability (**Table 2**).

**Table 2:** Summary of model performance indicators after 5 cross-validation.

Model	AUC	F1	Accuracy	Precision	Recall
Lasso	0.966 ± 0.016	0.839 ± 0.067	0.897 ± 0.037	0.867 ± 0.061	0.821 ± 0.111
SVM	0.952 ± 0.029	0.825 ± 0.082	0.893 ± 0.049	0.884 ± 0.088	0.787 ± 0.139
Logistic Regression	0.954 ± 0.046	0.823 ± 0.116	0.886 ± 0.067	0.838 ± 0.108	0.818 ± 0.161
Random Forest	0.914 ± 0.079	0.75 ± 0.127	0.846 ± 0.087	0.844 ± 0.17	0.679 ± 0.107
XGBoost	0.944 ± 0.045	0.832 ± 0.077	0.892 ± 0.047	0.862 ± 0.08	0.805 ± 0.078
GBM	0.95 ± 0.035	0.776 ± 0.109	0.855 ± 0.065	0.786 ± 0.092	0.776 ± 0.156

Additionally, model performance was evaluated using multiple random seeds, with trends remaining consistent across runs (**Supplementary Table 1**). Overall, the Lasso-based model consistently demonstrated high discriminative ability with low performance variability, suggesting relatively stable generalization across different data partitions. PDW, MPV, MCV, HCT, PCT, EOS%, MON#, MON%, RDW-CV, and NEU% were included in the Lasso-based model.

### 3.3 Clinical applicability of the diagnostic models

**Figure 4:**
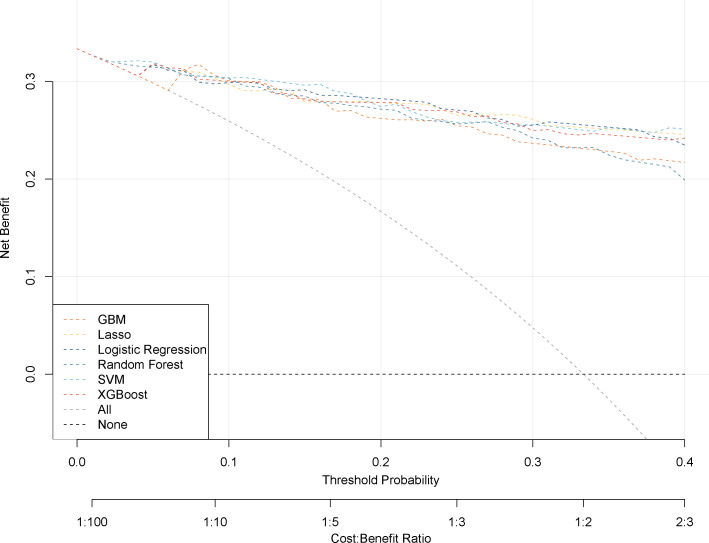
Decision curve analysis for six ML models. The x-axis represents the diagnostic probability threshold (0.0-0.4), and the y-axis represents net benefit (0-0.3). The cost-benefit ratio was set from 1:100 to 2:3. GBM (orange dashed line), Random Forest (teal dashed line), Lasso regression (yellow dashed line), Logistic Regression (dark blue dashed line), SVM (light blue dashed line), and XGBoost (red dashed line). Gray and black dashed lines represent the extreme strategies (“treat-all” and “treat-none”) as baselines.

DCA was performed to evaluate the clinical utility of the diagnostic models by quantifying net benefit (NB) across different threshold probabilities. The net benefit of each model was compared with two reference strategies: treat-all and treat-none (**Figure 4**). In these plots, the x-axis represents the threshold probability, and the y-axis represents net benefit, where NB > 0 indicates potential added value relative to a no-intervention strategy.

As shown in **Figure 4**, most diagnostic models demonstrated a higher net benefit than both the treat-all and treat-none strategies within the clinically relevant threshold probability range of 0–0.4. Specifically, the XGBoost, Lasso, and logistic regression models yielded positive net benefit between threshold probabilities of 0.05 and 0.4. The SVM and random forest models demonstrated positive net benefit from 0.01 to 0.4, while the GBM model showed positive net benefit between 0.07 and 0.4.

To provide clinical context, we considered a screening-oriented scenario in which “intervention” refers to referring PLWH for further psychiatric evaluation based on model predictions. The clinical benefit corresponds to early identification of true-positive schizophrenia cases, while the potential harm reflects false-positive predictions, which may lead to unnecessary referral, additional testing, medication exposure, psychological burden, social stigma, and economic costs. Threshold probability thus represents the clinician’s implicit trade-off between benefit and harm. Within this clinically relevant range, models with higher net benefit may provide supplementary decision support for mental health screening in PLWH.

### 3.4 Model interpretability analysis

**Figure 5:**
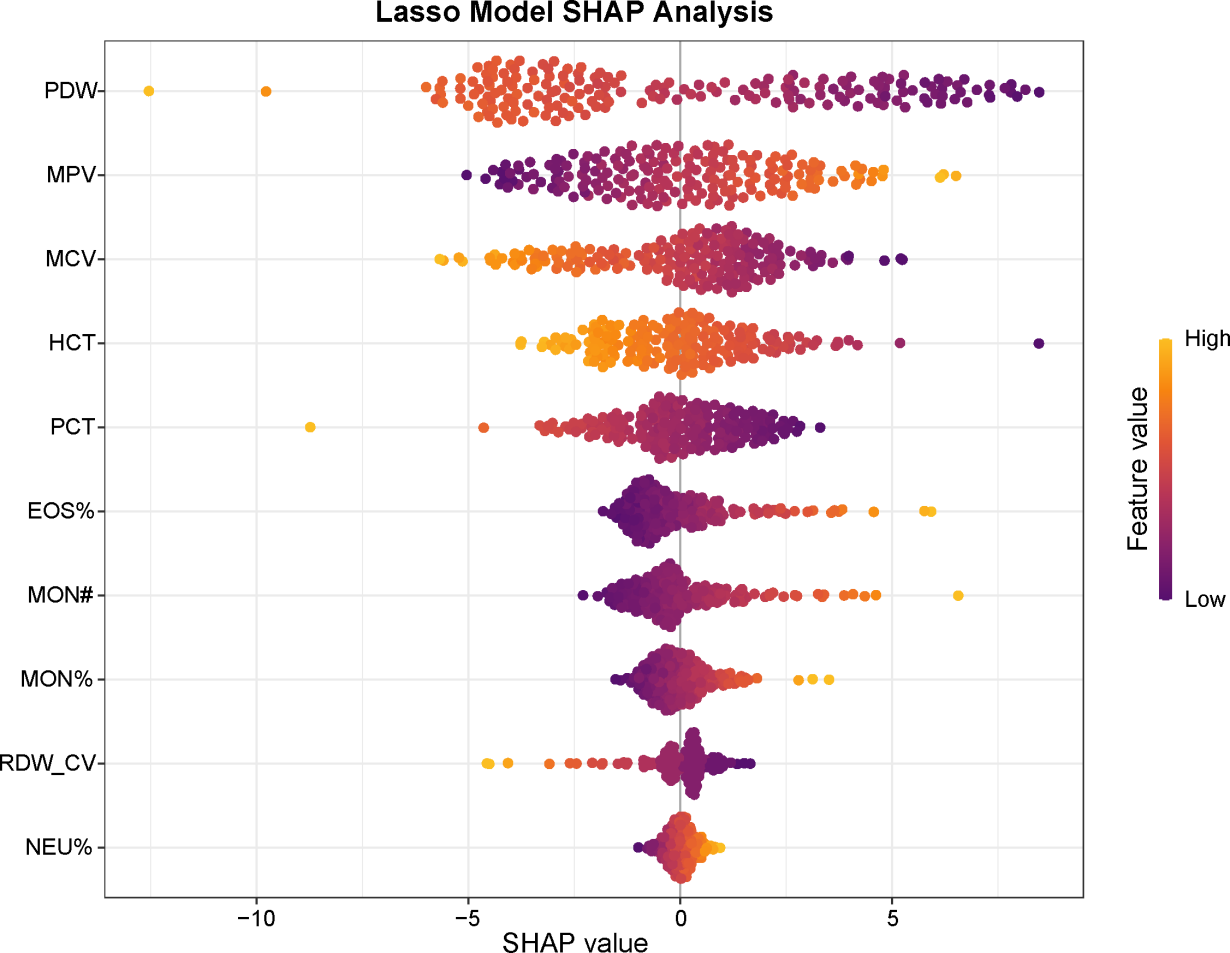
SHAP values and feature interaction scores in the Lasso model for schizophrenia diagnosis. Distribution of SHAP values showing each feature’s impact on model output. Feature values are color-coded: orange indicates higher values, and purple indicates lower values. Abbreviations: SHAP, SHapley Additive exPlanations; PDW, platelet distribution width; MPV, mean platelet volume; MCV, mean corpuscular volume; HCT, hematocrit; hematocrit; PCT, plateletcrit; EOS%, eosinophil percentage; MON#, absolute monocyte count; MON%, monocyte percentage; RDW-CV, red cell distribution width–coefficient of variation; NEU%, neutrophil percentage.

To improve interpretability, internal evaluation was performed to determine the relative importance of each variable in the diagnostic model. Shapley Additive Explanations (SHAP) analysis was applied to quantify feature contributions within the Lasso model (**Figure 5**). The results indicated that PDW, MPV, MCV, and HCT contributed most to the model’s predictions, followed by PCT, EOS%, MON, MON%, RDW-CV, and NEU%.

## 4 Discussion

In this study, we developed and internally validated machine learning–based models to identify schizophrenia among PLWH using routine hematological parameters. Our results indicate that, within an analytical framework incorporating 28 routine hematological parameters, all six machine learning models demonstrated acceptable discriminatory performance, with the Lasso model achieving the most favorable overall results. DCA further suggested that the model may provide a higher net clinical benefit across a range of clinically relevant threshold probabilities.

From a clinical decision-making perspective, the potential benefit of correct intervention is reflected in true-positive predictions, whereby patients with schizophrenia are accurately identified and may receive timely treatment, potentially mitigating disease progression and functional deterioration. Conversely, the cost of incorrect intervention arises from false-positive predictions, which may expose individuals without schizophrenia to unnecessary diagnostic labeling, additional examinations, medication-related adverse effects, psychological burden, social stigma, and economic costs. In real-world settings, the principal value of the proposed model lies in its ability to facilitate early identification of high-risk schizophrenia cases that might otherwise be overlooked by routine clinical assessment, thereby enabling earlier referral and intervention. Nevertheless, its potential harm is mainly associated with false-positive predictions, underscoring the need to balance clinical benefit against overtreatment. DCA provides a quantitative framework for this trade-off and indicates that the model yields a favorable net benefit within clinically relevant probability thresholds.

SHAP analysis further identified PDW, MPV, MCV, and HCT as the key features contributing most to model predictions. Although PCT did not show a significant difference between groups, its high contribution in the model suggests potential predictive value through complex interactions, which may be closely related to the pathophysiological processes of schizophrenia in the PLWH population. Our findings underscore the important role of platelet-related parameters in schizophrenia. PDW, reflecting platelet size variability and reactivity, was significantly reduced in the HIV-Scz group and emerged as the most critical predictor in SHAP analysis. Changes in platelet activation status may reflect peripheral immune dysregulation and neuroinflammatory processes, which are implicated in the pathophysiology of psychiatric disorders (28). In the HIV-Scz group, PDW and MCV were significantly decreased, PCT remained stable, and MPV showed a non-significant increasing trend, suggesting alterations in platelet volume–related parameters without evidence of overt global platelet activation. Furthermore, inflammatory ratio indices such as PLR and NLR did not differ significantly, indicating that hematological changes in this population may reflect a state characterized by selective platelet parameter alterations and immune imbalance rather than acute systemic inflammation.

MPV is a commonly used indicator of platelet activity, and reports of platelet- and inflammation-related indices in schizophrenia patients have been inconsistent. For example, Qi Yu et al. observed increases in PLT, MPV, P-LCR, PDW, PCT, and inflammatory ratios (NLR, PLR, MLR) in first-episode acute schizophrenia patients, suggesting enhanced acute inflammatory response and platelet reactivity (29). Whereas Behice Han Almış et al. reported decreased MPV and PCT in chronic schizophrenia patients, potentially reflecting platelet exhaustion or the effects of long-term antipsychotic treatment (30).

In our HIV-Scz cohort, MPV showed a non-significant upward trend, PDW was significantly decreased, PCT remained stable, and NEU% showed no significant change. This mixed phenotype suggests that in PLWH with schizophrenia, platelet- and hematology-related parameters may be influenced both by inflammatory regulation and chronic immune alterations, although the precise mechanisms remain to be elucidated.

In this study, MCV levels were significantly decreased in the HIV-Scz group. In contrast, Ransing et al. reported that schizophrenia patients generally exhibit higher MCV than healthy controls, regardless of antipsychotic treatment (31). This discrepancy may reflect the effects of chronic immune activation, inflammation-mediated bone marrow suppression, nutritional and metabolic abnormalities, and long-term antiretroviral therapy (ART) associated with HIV infection on erythropoiesis. Overall, the observed reduction in MCV in our cohort may represent a combined effect of HIV-related immune dysregulation and schizophrenia-related pathophysiological processes, highlighting the need to consider the specific immune and metabolic background when interpreting hematological parameters.

Consistent with previous studies, the HIV-Scz group exhibited a significant increase in monocyte percentage (MON%), suggesting that monocytes may play a key role in HIV-associated immune activation and central nervous system inflammation. Mazza et al. proposed that monocyte counts can serve as an indirect marker of microglial activation in the CNS (32), while Zhou et al. reported associations between monocyte counts, the monocyte-to-lymphocyte ratio (MLR), and cognitive deficits in schizophrenia patients (33). In this study, EOS% was also significantly elevated, indicating a potential imbalance in immune cell subpopulations, which may reflect chronic inflammation or the effects of pharmacological treatment on the immune system (34, 35). Notably, composite inflammatory indices such as PLR, NLR, and SII did not show concurrent increases, which may reflect HIV-specific immune alterations or a distinct immune response to comorbid psychiatric disorders rather than typical systemic inflammation (36–40). Taken together, these findings suggest that the immune-related hematological profile of schizophrenia in PLWH may differ from that observed in schizophrenia alone, and the underlying mechanisms require further investigation while accounting for key variables such as HIV disease stage and treatment regimens.

To our knowledge, this is the first study to apply routine hematological parameters combined with machine learning to identify schizophrenia among PLWH. We acknowledge that schizophrenia is inherently associated with immune and inflammatory abnormalities, and that its treatment with antipsychotic medications can affect hematopoiesis and immune function, potentially influencing hematological parameters (41, 42). Despite these multifactorial influences, the models developed in this study demonstrated potential utility within the current research framework, offering a low-cost and scalable tool for auxiliary screening or diagnostic purposes in resource-limited settings. Moreover, our findings highlight the potential of AI-driven models to integrate multidimensional clinical data for precision diagnostics. By identifying key predictive features, these models may provide insights for future investigations into pathophysiological mechanisms or targeted intervention strategies, although the underlying mechanisms require validation in subsequent studies.

Nonetheless, several limitations should be acknowledged. First, the retrospective single-center design of this study limits causal inference and may introduce selection bias, thereby affecting the generalizability of the findings. Second, although the models were evaluated using five-fold cross-validation, the overall sample size remains relatively modest. Therefore, their external generalizability, particularly across different regions or populations, warrants further validation. In addition, all schizophrenia cases included in this study were clinically confirmed and receiving antipsychotic treatment at the time of blood sampling. As a result, the observed alterations in hematological parameters and the corresponding model performance may reflect a combination of disease-related and treatment-associated effects.

Moreover, hematological parameters may be influenced by ART, antipsychotic medications, and comorbid conditions, which are potential confounders that cannot be fully disentangled within the current study design. Future validation in multicenter, prospective cohorts—especially among treatment-naïve populations—will be necessary to clarify the relative contributions of the disease itself versus treatment-related factors. Finally, although the models demonstrated good interpretability and potential utility at the population level, their specific role in supporting individual-level clinical decision-making requires further evaluation in real-world clinical settings.

## 5 Conclusion

In conclusion, this study demonstrates that AI-driven models based on routine hematological parameters exhibit good predictive performance for identifying schizophrenia risk among PLWH. PDW, MCV, MPV, and PCT emerged as the most important predictive features, reflecting underlying immune, inflammatory, and erythroid alterations. These models provide a practical and low-cost tool for early detection and have the potential to support improved clinical management in this high-risk population.

## Data Availability

This study is based on retrospective data from a single center with a limited sample size, which may affect generalizability. Reliance on medical records may introduce missing or incomplete information, and hematological parameters were obtained using one analyzer type, potentially limiting comparability. Unmeasured confounders such as medication use, nutritional status, or co-infections could also influence results. Requests to access these datasets should be directed to YL, youwanglu@163.com.

## Glossary

AI: artificial intelligence
ART: antiretroviral therapy
AUC: Area Under the Curve
BAS%: basophil percentage
BAS#: absolute basophil count
CBC: complete Blood Count
DCA: decision curve analysis
EOS#: absolute eosinophil count
EOS%: eosinophil percentage
HCT: hematocrit
HIV: Human Immunodeficiency Virus
HIV-Scz: HIV associated schizophrenia
HGB: hemoglobin
IQR: interquartile range
Lasso: least absolute shrinkage and selection operator
GBM: gradient boosting machine
LYM%: lymphocyte percentage
LYM#: absolute lymphocyte count
MCH: mean corpuscular hemoglobin
MCHC: mean corpuscular hemoglobin concentration
MCV: mean corpuscular volume
ML: machine learning
MON#: absolute monocyte count
MON%: monocyte percentage
mRNA: examined messenger RNA
MLR: monocyte-to-lymphocyte ratio
MRI: magnetic resonance imaging
MPV: mean platelet volume
NEU%: neutrophil percentage
NEU#: absolute neutrophil count
NLR: neutrophil-to-lymphocyte ratio
PAM: prediction analysis for microarrays
PCT: plateletcrit
PDW: platelet distribution width
PLT: platelet count
PLR: platelet-to-lymphocyte ratio
PLWH: people living with HIV
RBC: red blood cell count
RDW-CV: red cell distribution width–coefficient of variation
RDW-SD: red cell distribution width–standard deviation
ROC: receiver operating characteristic
SD: standard deviation
SII: systemic immune-inflammation index
SIRI: systemic inflammation response index
SHAP: Shapley additive explanations
SVM: support vector machine
WBC: white blood cell count
XGBoost: extreme gradient boosting.
