# Supplementary material for: Artificial intelligence-based diagnostic model for schizophrenia in individuals living with HIV
Source: Front Psychiatry. 2026 Mar 9;17:1709861. doi: 10.3389/fpsyt.2026.1709861 (PMC13006588; doi:10.3389/fpsyt.2026.1709861)
Supplement: Supplementary file 1 [file Table1.docx]

| **Model** | **Seed** | **AUC** | **F1-score** | **Accuracy** | **Precision** | **Recall** |
| --- | --- | --- | --- | --- | --- | --- |
| Lasso​ | 2 | 0.958±0.03 | 0.845±0.096 | 0.892±0.079 | 0.856±0.158 | 0.855±0.105 |
|  | 33 | 0.973±0.021 | 0.883±0.053 | 0.919±0.042 | 0.884±0.093 | 0.887±0.044 |
|  | 123 | 0.959±0.035 | 0.824±0.128 | 0.887±0.075 | 0.84±0.123 | 0.822±0.176 |
|  | 456 | 0.963±0.035 | 0.829±0.093 | 0.892±0.055 | 0.849±0.068 | 0.818±0.138 |
|  | 2026 | 0.968±0.024 | 0.865±0.073 | 0.914±0.04 | 0.885±0.067 | 0.853±0.111 |
| SVM​ | 2 | 0.976±0.019 | 0.792±0.055 | 0.876±0.024 | 0.91±0.103 | 0.724±0.147 |
|  | 33 | 0.948±0.023 | 0.788±0.047 | 0.866±0.026 | 0.827±0.051 | 0.758±0.081 |
|  | 123 | 0.947±0.023 | 0.794±0.07 | 0.866±0.047 | 0.823±0.106 | 0.773±0.072 |
|  | 456 | 0.962±0.016 | 0.808±0.065 | 0.881±0.032 | 0.867±0.083 | 0.774±0.147 |
|  | 2026 | 0.956±0.027 | 0.821±0.075 | 0.887±0.037 | 0.859±0.086 | 0.808±0.16 |
| Random Forest​ | 2 | 0.93±0.016 | 0.734±0.07 | 0.839±0.027 | 0.822±0.118 | 0.691±0.158 |
|  | 33 | 0.926±0.017 | 0.742±0.054 | 0.838±0.028 | 0.793±0.063 | 0.706±0.102 |
|  | 123 | 0.913±0.047 | 0.724±0.09 | 0.828±0.056 | 0.784±0.106 | 0.679±0.107 |
|  | 456 | 0.919±0.06 | 0.726±0.146 | 0.833±0.073 | 0.77±0.095 | 0.708±0.215 |
|  | 2026 | 0.937±0.024 | 0.748±0.044 | 0.85±0.03 | 0.87±0.113 | 0.676±0.118 |
| XGBoost​ | 2 | 0.936±0.053 | 0.758±0.123 | 0.85±0.072 | 0.806±0.103 | 0.727±0.163 |
|  | 33 | 0.939±0.047 | 0.795±0.058 | 0.866±0.042 | 0.821±0.087 | 0.777±0.077 |
|  | 123 | 0.943±0.017 | 0.746±0.048 | 0.839±0.018 | 0.79±0.069 | 0.728±0.143 |
|  | 456 | 0.937±0.042 | 0.761±0.114 | 0.844±0.07 | 0.78±0.107 | 0.756±0.146 |
|  | 2026 | 0.927±0.031 | 0.78±0.059 | 0.86±0.035 | 0.832±0.11 | 0.756±0.134 |
| GBM | 2 | 0.951±0.029 | 0.818±0.042 | 0.882±0.024 | 0.841±0.057 | 0.806±0.094 |
|  | 33 | 0.943±0.02 | 0.794±0.044 | 0.861±0.032 | 0.784±0.047 | 0.808±0.069 |
|  | 123 | 0.952±0.036 | 0.802±0.108 | 0.871±0.068 | 0.82±0.091 | 0.795±0.159 |
|  | 456 | 0.936±0.028 | 0.755±0.091 | 0.844±0.057 | 0.794±0.106 | 0.726±0.106 |
|  | 2026 | 0.943±0.041 | 0.766±0.118 | 0.848±0.071 | 0.768±0.085 | 0.769±0.162 |
| Logistic Regression | 2 | 0.97±0.02 | 0.868±0.049 | 0.914±0.035 | 0.907±0.098 | 0.838±0.055 |
|  | 33 | 0.962±0.038 | 0.844±0.068 | 0.898±0.044 | 0.869±0.097 | 0.842±0.143 |
|  | 123 | 0.958±0.032 | 0.831±0.045 | 0.887±0.034 | 0.846±0.094 | 0.823±0.066 |
|  | 456 | 0.96±0.03 | 0.818±0.061 | 0.882±0.044 | 0.866±0.13 | 0.788±0.079 |
|  | 2026 | 0.97±0.021 | 0.85±0.068 | 0.904±0.044 | 0.897±0.115 | 0.823±0.107 |

**Supplementary Table 1.** Performance of six machine learning models under different random seeds.
